# Social capital and dental pain in Brazilian northeast: a multilevel cross-sectional study

**DOI:** 10.1186/1472-6831-13-2

**Published:** 2013-01-04

**Authors:** Bianca Marques Santiago, Ana Maria Gondim Valença, Mario Vianna Vettore

**Affiliations:** 1 Department of Clinic and Social Dentistry, Federal University of Paraíba, Rua Silvino Chaves, 1061 (ap.1401), Manaíra, João Pessoa, PB, CEP: 58.038-420, Brazil; 2 Department of Clinic and Social Dentistry, Federal University of Paraíba, Rua Jacinto Dantas, 94 (ap. 206), Manaíra, João Pessoa, PB, CEP: 58038-270, Brazil; 3 Institute of Studies in Public Health, Federal University of Rio de Janeiro, Av. Brigadeiro Trompowski, s/nº.Praca da Prefeitura, Rio de Janeiro/RJ, CEP 21949-900, Brazil

**Keywords:** Dental pain, Epidemiology, Oral health, Social capital, Socioeconomic factors

## Abstract

**Background:**

There is limited evidence on possible associations between social determinants and dental pain. This study investigated the relationship of neighborhood and individual social capital with dental pain in adolescents, adults and the elderly.

**Methods:**

A population-based multilevel study was conducted involving 624 subjects from 3 age groups: 15–19, 35–44 and 65–74 years. They were randomly selected from 30 census tracts in three cities in the State of Paraíba, Brazil. A two-stage cluster sampling was used considering census tracts and households as sampling units. The outcome of study was the presence of dental pain in the last 6 months. Information on dental pain, demographic, socio-economic, health-related behaviors, use of dental services, self-perceived oral health and social capital measures was collected through interviews. Participants underwent a clinical examination for assessment of dental caries. Neighborhood social capital was evaluated using aggregated measures of social trust, social control, empowerment, political efficacy and neighborhood safety. Individual social capital assessment included bonding and bridging social capital. Multilevel logistic regression was used to test the relationship of neighborhood and individual social capital with dental pain after sequential adjustment for covariates.

**Results:**

Individuals living in neighborhoods with high social capital were 52% less likely to report dental pain than those living in neighborhoods with low social capital (OR = 0.48, 95% CI = 0.27-0.85). Bonding social capital (positive interaction) was independently associated with dental pain (OR = 0.88, 95% CI = 0.80-0.91). Last dental visit, self-perceived oral health and number of decayed teeth were also significantly associated with dental pain.

**Conclusions:**

Our findings suggest that contextual and individual social capital are independently associated with dental pain.

## Background

Evidence in oral health research highlights the underlying influence of social, economic, environmental and political determinants that act via material, behavioral and psychosocial pathways [1] on oral diseases [2]. In particular, both compositional and contextual factors of where people live influence their health [3]. Compositional explanations attribute the effect to characteristics of the individuals, such as social status and social position. On the other hand, contextual explanations for local environment effects on health operate through broader psychosocial and material pathways. Moreover, material circumstances can have psychosocial consequences and vice versa [3,4]. Inequalities in oral health mirror those in general health [2]. It has been shown a social gradient in morbidity and mortality levels and also that its universality indicates the overriding influence of the social environment or social context on health [5]. According to Marmot ‘if the major determinants of health are social, so must be the remedies’ [6].

There are few epidemiological studies on the social determinants of dental pain. They predominantly address the relationship of a family’s social position, its socioeconomic status and cumulative episodes of poverty to children’s dental pain [7-9] and to adult’s orofacial and dental pain [10]. Only one previous study reported the association between contextual socioeconomic status, namely the Human Development Index, and dental pain. Using a multilevel analysis, it was reported that dental pain was 33% less prevalent in adolescents living in more developed areas of the city, compared to those from less developed ones [11].

The concept of social capital has entered into the mainstream of public health discourse since the 1990s, representing a new branch of studies of social determinants of health [12]. There is no consensus on the definition and measurement of social capital. However, the majority of concepts of social capital assume that it consists of some aspects of social structure and facilitates certain actions of individuals who are within the structure [13]. Social capital can be defined in terms of resources, such as the levels of social support and social information that are embedded within an individual’s social network. In contrast, others conceptualize and measure social capital as both an individual attribute as well as a property of the collective [12,14]. Most studies did not simultaneously assess the relationship between social capital and health at both individual and group levels, but tended to assess one or the other [12].

There is evidence on the possible effect of social capital on oral health. Previous studies focused on adolescents or elderly people limiting their findings to specific age groups. Studies on social capital and oral health considered clinical and subjective measures of oral health including dental caries, dental injuries, number of remaining teeth and self-rated oral health [15-19]. In addition, social capital has been assessed at individual and community levels.

In the studies where social capital was considered as a community-level characteristic, the most common measurement of social capital was aggregating data at individual level. Neighborhood social capital was assessed using a 30-item social capital index including five dimensions of social capital (social trust, social control, empowerment, neighborhood security and political efficacy) in a study on social capital and dental injuries in Brazilian adolescents [15]. In another study, vertical and horizontal social capital were considered community-level variables that were calculated using the average scores of individuals nested in communities. Horizontal social capital showed beneficial effects on numbers of remaining teeth in older Japanese adults [20]. In two other studies in social capital and oral health in Japan, community-level social capital was created by aggregating individual-level data [17,18]. Individuals aged 65 years or over living in communities with higher structural social capital (volunteer participation) reported more number of natural teeth [17]. In the other study, neighborhood social capital (social network) was independently associated with individual dentate status regardless of individual social networks and social support [18].

**Figure 1:**
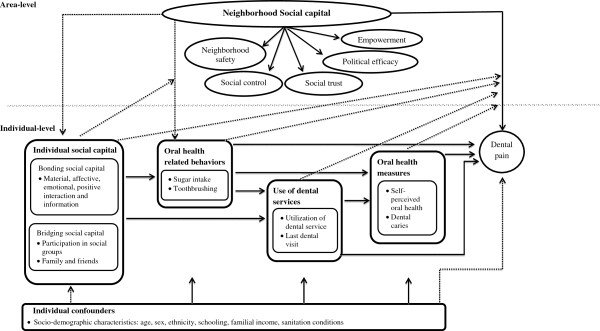
Conceptual model of neighborhood and individual social capital on dental pain.

No study has explored the association between social capital at individual and community level and dental pain. A theoretical framework based on Carpiano study [21] was developed to test this association and is presented in Figure 1. Neighborhood social capital was the second-level area variable, which exert a direct influence on the occurrence of dental pain. Otherwise, the link between neighborhood social capital and dental pain can be mediated through individual social capital and oral health related behaviors. Individual level variables were individual social capital, oral health related behaviors, use of dental services and oral health measures. Individual social capital can affect oral health related behaviors and use of dental services and, therefore, influence dental pain. Oral health related behaviors, use of dental services and oral health measures are interconnected and are considered proximal determinants of dental pain. Potential confounders were socio-demographic characteristics. Therefore, this study tested the relationship of neighborhood and individual social capital with dental pain in adolescents, adults and elderly.

## Methods

The Human Research Ethics Committee of the Department of Health of the State of Paraíba gave ethical approval for the study (Protocol no. 0001.0.349.000-09) and informed consent was obtained from all participants or their parents in case of adolescents under 18 years.

### Design and sample

Data were obtained from a multilevel population-based study conducted between 2010 and 2011 in three cities in the State of Paraíba, Northeast Brazil. A household survey with two stage sampling method was conducted. First, a random sample of 30 neighborhoods, among the 76 eligible, was selected. Then, adolescents, adults and elderly living in the neighborhoods were randomly selected from the official record of local health services. Census tracts are administrative boundaries that represent characteristics of similar communities with a similar population density and which in the present study were considered as neighborhoods. The sample of adolescents, adults and elderly was proportionally selected across the census tracts. As a result, the proportion of age groups in the sample was similar among the selected areas. Inclusion criterion for participants was age between 15 and 19 years (adolescents), or between 35 and 44 years (adults), or between 65 and 74 years (elderly). The exclusion criteria were living outside the selected neighborhoods and individuals under orthodontic treatment, since pain is common during orthodontic treatment.

A minimum sample size of 593 people, proportionally selected from 30 neighborhoods, was estimated; assuming a significance level of 5%, an 85% power, design effect of 1.5 and a prevalence of dental pain in people aged ≥ 15 years of 25% in neighborhoods with high social capital and 35% in neighborhoods with low social capital (10% difference) [22].

### Data collection

Initially 30 neighborhoods (census tracts) were randomly selected. Thereafter, residents were invited to participate in the study to obtain individual data through face-to-face interviews at participants’ households. Dental caries assessment was conducted by three examiners previously calibrated using the DMFT index. Intra-examiner and inter-examiner Kappa Coefficient were ≥ 0.93 and Kappa ≥ 0.89, respectively.

### Outcome variable

The outcome of study was the prevalence of reported dental pain in the last 6 months. Participants were asked the following question: “Have you had toothache during the last six months?” with options “yes” or “no”. Therefore, a binary outcome variable was used in the multilevel logistic regression analysis. This variable was used in previous studies [9,11].

### Contextual social capital

Contextual social capital was defined as the features of social organization; such as civic participation, norms of reciprocity, and trust in others; that facilitate cooperation for mutual benefit [12]. The questionnaire used to measure contextual social capital included 30 items comprising 5 dimensions. Social trust dimension was composed by 9 items, 5 items with 3 options (0 to 2) and 4 items with 5 options (0 to 4). The score of social trust dimension ranged from 0 to 26 [23]. Social control dimension included 5 items with 3 options (0 to 2) with score ranging from 0 to 10 [24]. Five items with 5 options (0 to 4) were used in the Empowerment dimension, which score ranged from 0 to 20 [25]. Political efficacy dimension was composed by 4 items with 3 options (0 to 2), score from 0 to 8 [26]. Neighborhood safety dimension was composed by 4 items with 3 options (0 to 2), score from 0 to 8 [23]. Because of the differing numbers of items in each subscale (dimension), the final score of each sub-scale was standardized from 0 to 100 points. The score obtained in each dimension was multiplied by 100 divided and by the maximum score. For example, a participant who answered the intermediate value (1) in each item of the political efficacy dimension (4 items) would have a final score of 4. The standardized score of political efficacy dimension was [4 × 100]/8 = 50. The final score was an unweighted sum of each subscale. The social capital questionnaire was previously developed and tested in a Brazilian sample with an adequate internal consistency (Cronbach’s α coefficient > 0.70) [15].

The questions on social capital were collected individually and then aggregated at a neighborhood level [12], assuming that questions are related to neighborhood. This categorization was used previously [15]. The 30 neighborhoods were divided into three levels: low, intermediate and high social capital areas based on the tertiles of the mean score of social capital [15,18]. The social capital at neighborhood level was used as a secondary-level variable.

### Individual social capital – bonding and bridging social capital

Bonding social capital involves having strong ties with people in the same community that enable people to ‘get by’. It is characterized by a flow of information and support among members of a particular group [27]. Bonding was assessed by using a social support scale consisting of 19 items comprising 5 dimensions of functional support: material, affective, emotional, positive social interaction and information [28,29].

Bridging social capital is the formal and informal links with other community members that enables people to ‘get ahead’. Bridging connects individuals and groups, corresponding to people’s social networks, and enables an information and resource flow between the groups [27]. The social network questionnaire consisted of five questions concerning the person’s relationship with their family and friends, and their participation in social groups [29].

### Confounders and mediators

Demographic and socioeconomic confounders included sex, age, ethnicity, schooling, familial income and sanitation conditions. Oral health-related behaviors (frequency of sweet intake and tooth brushing), use of dental services, self-perceived oral health and number of decayed teeth were considered mediators, because they are likely to be on the pathway between social capital and dental pain.

### Statistical analysis

A multilevel logistic model was used to estimate the association between contextual social capital (an area-level variable), individual social capital (bonding and bridging social capital) and dental pain, controlling for potential confounders and mediators according to the theoretical framework presented in Figure 1.

Statistical modeling was initially carried out by bivariate analysis in order to select relevant independent variables described in Figure 1. Only covariates presenting *P* < 0.10 were considered in multilevel models. This criterion was used to reduce discrepancy between the data and the model and reach an economic model with relatively few parameters. Co-linearity was detected among the five dimensions of bonding social capital. Positive interaction was the bonding social capital dimension elected for multivariate analysis because it was the one that showed higher statistical significance.

A two-level random-intercepts and fixed-slopes model structure with individuals nested within neighborhoods was fitted and used to estimate the cumulative distribution probabilities of the two groups being compared. The fixed and random-parameter estimates for the two-level ordered *logit* models were calculated by predictive/penalized *quasi*-likelihood (PQL) procedures with second-order Taylor series expansion.

The unadjusted association of social capital (Model 1) was sequentially adjusted for bonding and bridging social capital (compositional effect) in Model 2, individual-level confounders in Model 3 (socio-demographic characteristics), and individual-level mediators in Model 4 (use of dental services) and Model 5 (self-perceived oral health and number of decayed teeth). The entrance of the independent variables across the statistical modeling was theoretically driven based on the conceptual framework (Figure 1). The significance level established for multilevel analysis was 5% (*P* ≤ 0.05).

All statistical analyses were conducted using SPSS 17.0 (Statistical Package for the Social Sciences for Windows®, SPSS Inc., Chicago, IL, USA), and MLwiN software version 2.24 (Centre for Multilevel Modeling, Bristol, UK).

## Results

Of 763 individuals invited to participate, 661 agreed to take part in the study (response rate = 86.3%). Participants with missing data for the outcome or any independent variable used in multilevel analysis were excluded (N = 37), therefore, 624 subjects composed the final analytical sample.

The individual characteristics of the sample and unadjusted associations between first level independent variables and dental pain are presented in Table 1. The overall prevalence of dental pain was 26.8%, ranging from 0 to 44.4% between the selected neighborhoods. The sample was predominantly composed of adolescents (60.1%) and females (62.8%). Categories of family income were defined according to Brazilian minimum wage and converted into American dollars. Unadjusted associations at a 10% significance level of socio-demographic characteristics were observed between age, sex, years of schooling and dental pain. Although none of the oral health-related behaviors were associated with dental pain, all dental services variables and oral health measures showed significant associations with dental pain (Table 1).

**Table 1:** Estimated unadjusted Odds Ratios (OR) from first level variables for dental pain

	**Dental Pain**				
	** Yes**	** No**	**OR**	95% CI*	***P***
	**n = 167**	**n = 457**			
***Socio-demographic characteristics***					
Age group, n (%)					
Adolescents (15–19 years)	106 (63.5)	269 (58.9)	1		
Adults (35–44 years)	52 (31.1)	143 (31.3)	0.92	0.62-1.32	0.686
Elderly (65–74 years)	9 (5.4)	45 (9.8)	0.51	0.24-1.07	0.076
Sex, n (%)					
Male	52 (31.1)	180 (39.4)	1		
Female	115 (68.9)	277 (60.6)	1.44	0.98-2.10	0.060
Ethnicity, n (%)					
White	39 (23.4)	113 (24.7)	1		
Brown	118 (70.6)	313 (68.5)	1.09	0.72-1.66	0.681
Black	10 (6.0)	31 (6.8)	0.93	0.42-2.08	0.869
Years of schooling, n (%)					
≥ 9	48 (28.8)	177 (38.7)	1		
5-8	74 (44.3)	176 (38.5)	1.55	1.02-2.36	0.040
≤ 4	45 (26.9)	104 (22.8)	1.60	0.99-2.56	0.053
Family Income, n (%)^a^					
≤ $430	11 (7.5)	37 (9.5)	1		
$431-860	50 (34.2)	95 (24.4)	1.77	0.83-3.77	0.138
$861 to 2,580	74 (50.8)	205 (52.5)	1.21	0.59-2.50	0.599
> $ 2,580	11 (7.5)	53 (13.6)	0.70	0.27-1.78	0.451
Sanitation conditions, n (%)^b^					
Water supply inside house	147 (88.6)	415 (91.0)	1		
No water supply/outside house	19 (11.4)	41 (9.0)	1.31	0.74-2.33	0.360
***Oral health-related behaviors***					
Frequency of sweet intake, n(%)^c^					
Never	22 (13.3)	57 (12.5)	1		
1-3 days per week	86 (51.8)	231 (50.9)	0.97	0.56-1.67	0.898
≥ 4 days per week	58 (34.9)	166 (36.6)	0.91	0.51-1.61	0.735
Frequency of tooth brushing, n(%)					
≥ 3 times per day	91 (54.5)	288 (63.0)	1		
2 times per day	60 (35.9)	139 (30.4)	1.36	0.93-2.01	0.111
1 time per day	16 (9.6)	30 (6.6)	1.69	0.88-3.24	0.115
***Use of dental services***					
Utilization of dental care, n(%)					
No	3 (1.8)	19 (4.4)	1		
Yes	164 (98.2)	437 (95.6)	3.78	0.87-16.33	0.075
Last dental visit, n (%)					
<1 year	110 (65.9)	235 (51.4)	1		
≥1 years	54 (32.3)	203 (44.4)	0.57	0.39-0.83	0.003
Never	3 (1.8)	19 (4.2)	0.34	0.10-1.16	0.085
***Oral health measures***					
Self-perceived oral health, n(%)					
Excellent/Good	31 (18.6)	175 (38.3)	1		
Fair/Poor/Very Poor	136 (81.4)	282 (61.7)	2.72	1.76-4.20	<0.001
N decayed teeth, mean ± SD	5.5 (4.5)	2.5 (3.2)	1.22	1.16-1.29	<0.001

^*^95% Confidence Interval; ^a^*n* = 532; ^b^*n* = 622; ^c^*n* = 620.

$430 = 1 Brazilian Minimal Wage.

The distribution of dental pain groups according to social capital variables is presented in Table 2. Unadjusted inverse associations were observed between high neighborhood social capital, bonding social capital, bridging social capital (relative social networks) and dental pain.

**Table 2:** Social capital variables and unadjusted multilevel Odds Ratios (OR) for dental pain

	**Dental pain**				
	** Yes**	** No**	**OR**	95% CI*	***P***
	** n = 167**	** n = 457**			
Neighborhood social capital, n (%)					
Low	64 (38.3)	145 (31.7)	1		
Moderate	60 (35.9)	154 (33.7)	0.88	0.58-1.34	0.559
High	43 (25.8)	158 (34.6)	0.62	0.39-0.96	0.034
Bonding social capital^a^, mean ± SD					
Affective Support	90.9 (14.7)	93.1 (13.8)	0.90	0.80-1.02	0.096
Emotional Support	82.1 (20.3)	85.1 (19.3)	0.93	0.85-1.01	0.093
Information Support	83.7 (20.1)	87.6 (17.7)	0.90	0.81-0.99	0.025
Positive Interaction	84.1 (19.0)	88.8 (16.6)	0.86	0.78-0.95	0.003
Material Support	88.4 (14.2)	89.6 (15.5)	0.95	0.85-1.07	0.401
Bridging social capital, n (%)					
Sport/artistic activities in the last year					
≥ 1	75 (44.9)	223 (48.8)	1		
0	92 (55.1)	234 (51.2)	1.17	0.82-1.67	0.390
Meetings in the last year					
≥ 1	23 (13.8)	52 (11.4)	1		
0	144 (86.2)	405 (88.6)	0.80	0.48-1.36	0.416
Charity work in the last year^b^					
≥ 1	28 (16.9)	102 (22.4)	1		
0	138 (83.1)	354 (77.6)	1.42	0.90-12.25	0.137
Relatives					
≥ 1	147 (88.0)	422 (92.3)	1		
0	20 (12.0)	35 (7.7)	1.64	0.92-2.93	0.095
Friends^b^					
≥ 1	110 (66.3)	316 (69.3)	1		
0	56 (33.7)	140 (30.7)	1.15	0.79-1.68	0.472

^*^95% Confidence Interval; ^a^Bonding social capital: OR estimates assessed by 10-points increase.

^b^*n* = 622.

The results of the multilevel logistic analysis between social capital and dental pain are shown in Table 3. In the unadjusted model (Model 1), the odds of dental pain were lower in the areas with high levels of neighborhood social capital. The second model (Model 2) presents the independent association of neighborhood social capital and individual social capital with dental pain. Individuals with high bonding social capital and those living in the areas with high neighborhood social capital showed lower odds of dental pain. Additional adjustments were conducted for individual socio-demographic characteristics (Model 3), use of dental care services (Model 4) and oral health related behaviors (Model 5). Bonding social capital and high neighborhood social capital remained inversely associated with dental pain across the models. Elderly people showed lower odds of dental pain compared to adolescents in Model 3. Furthermore, female sex and low schooling increased the odds of dental pain. In Model 4, age group and schooling remained independently associated with dental pain. In addition, the time since last dental visit was associated with dental pain.

**Table 3:** Multilevel logistic regression of the association between social capital and dental pain

	** Model 1**^**a**^	** Model 2**^**b**^	** Model 3**^**c**^	** Model 4**^**d**^	** Model 5**^**e**^
**Explanatory variables**	** OR (95% CI)**	** OR (95% CI)**	** OR (95% CI)**	** OR (95% CI)**	** OR (95% CI)**
***Individual-level variables***					
Age group (Reference: Adolescents)					
Adults			0.70 (0.44-1.11)	0.69 (0.43-1.10)	0.79 (0.47-1.33)
Elderly			**0.31 (0.13-0.72)**	**0.33 (0.14-0.80)**	0.81 (0.30-2.15)
Sex (Reference: Male)					
Female			**1.48 (1.00-2.21)**	1.34 (0.89-2.02)	1.53 (0.97-2.41)
Years of schooling (Reference: ≥ 9)					
5-8			1.68 (1.09-2.58)	1.94 (1.25-3.03)	1.45 (0.89-2.37)
≤ 4			**2.37 (1.35-4.17)**	**2.70 (1.51-4.81)**	1.33 (0.69-2.57)
Utilization of dental care (Reference: No)					
Yes				3.61 (0.46-28.22)	5.69 (0.49-66.1)
Last dental visit (Reference: <1 year)					
≥ 1 year(s)				**0.57 (0.38-0.86)**	**0.46 (0.29-0.72)**
Never				0.55 (0.09-3.37)	0.34 (0.04-2.61)
Self-perceived oral health (Reference: Excellent/good)					
Fair/Poor/Very Poor					**1.93 (1.18-3.15)**
Number of decayed teeth					**1.24 (1.16-1.31)**
	***Individual-level social capital***					
Bonding/Positive Interaction^f^		**0.87 (0.82-0.90)**	**0.88 (0.82-0.92)**	**0.88 (0.82-0.90)**	**0.88 (0.80-0.91)**	
Bridging/Relatives (Reference: ≥ 1)						
No		1.53 (0.84-2.77)	1.50 (0.81-2.75)	1.54 (0.83-2.84)	1.60 (0.81-3.16)	
***Neighborhood-level social capital*** (Reference: Low)						
Moderate social capital	0.88 (0.58-1.34)	0.87 (0.57-1.33)	0.85 (0.55-1.30)	0.82 (0.53-1.28)	0.73 (0.42-1.26)	
High social capital	**0.62 (0.39-0.96)**	**0.61 (0.39-0.96)**	**0.59 (0.37-0.93)**	**0.57 (0.35-0.90)**	**0.48 (0.27-0.85)**	

^**a**^Model 1 Unadjusted; ^**b**^Model 2, Model 1 plus adjustment for Individual-level social capital variables; ^**c**^Model 3, Model 2 plus adjustment for socio-demographic confounders (age group, sex, schooling); ^**d**^Model 4, Model 3 plus adjustment for dental services mediators (use of dental services, last dental visit), ^e^Model 5, Model 4 plus adjustment for oral health mediators (self-perceived oral health, number of decayed teeth) ^f^Bonding social capital: OR estimates assessed by 10-points increase.

According to the final model (Model 5), individuals living in neighborhoods with high social capital were 52% less likely to report dental pain than those living in neighborhoods with low social capital (OR, 0.48; 95% CI, 0.27-0.85). In addition, the odds of reporting dental pain was 12% lower among those with bonding social capital (positive social interaction) (OR, 0.88; 95% CI, 0.80-0.91). Individual factors associated with dental pain were the last dental visit, self-perceived oral health and number of decayed teeth (Table 3). Poor self-perceived oral health and dental caries increased the odds of dental pain.

## Discussion

In this study, the hypothesis that neighborhood and individual social capital are associated factors with dental pain was confirmed. Furthermore, neighborhood social capital showed a strongest association with dental pain compared to individual social capital. The odds of dental pain were 52% and 12% lower in those living in neighborhoods with higher social capital and in those with individual social capital, respectively, suggesting that the effect of social context on dental pain is particularly more important than individual social relationships. Similar to previous studies on the subject, contextual social capital was associated with oral health, but to the author’s knowledge this is the first study to show evidence of the relationship between neighborhood and individual social capital and dental pain.

Whereas studies focused linking social capital to oral health outcomes were on the impact of neighborhood social capital on children’s oral health [15,16], more attention has recently been given to the possible simultaneous influence of contextual and individual social capital on dental status [17,18]. The study by Aida *et al.*[20] showed a significant association between community and individual social capital and oral health. That agrees with our findings on dental pain. The relationship between neighborhood and individual social capital and other types of pain has been demonstrated. For example, bonding social capital and not bridging social capital was associated with pain functioning and quality of life in patients with fibromyalgia [30]. Low individual social capital was also associated with poor health outcomes, including musculoskeletal disorders [31]. Similarly, Swedish adolescents from neighborhoods with high social capital reported more symptoms of musculoskeletal pain and psychosomatic symptoms compared to those from areas with low social capital [32].

Different mechanisms underlying the influence of social capital on health outcomes may explain our findings. First, individual and neighborhood social capital may benefit health by a positive influence on health-related behaviors and pattern of attending dental services [15,16,18,33]. Neighborhoods with high levels of social capital are characterized by shared norms and a general consensus about what constitutes appropriate health practices [33]. Or individual social capital can make more possible diffusion of health information between individuals and spread positive behavioral norms through the communities [15,16]. It can be hypothesized that low bonding social capital may lead to poor oral hygiene and unhealthy dietary habits as well as lower use of dental services and, consequently, predisposing individuals to a greater likelihood for more severe dental caries and so dental pain.

Second, high levels of neighborhood social capital may protect psychosocial health; resulting in less fear, stress and anxiety as well as increasing self-esteem [16,32,33] and personal oral health care [16,33]. Psychological distress has been associated with harmful behaviors, such as smoking and consumption of ‘comfort foods’ such as confectionary [34], which in turn may increase the risk for periodontal diseases and dental caries [20], two clinical conditions related to dental pain experience. Third, residents in neighborhoods with high social capital are more likely to participate in civic activities and political processes in order to secure health-promoting resources such as educational opportunities and better health services [21,33]. Moreover, individual social capital can increase civic participation through interpersonal political influence within communities to make more efficient use of local physical and financial resources [16,21,33].

The theoretical framework used in this study pointed out the mediator effects of oral health clinical measures and behaviors on the association between social capital and dental pain. Previous studies reported the positive relationship between low social capital and dental caries [16,35] and poor self-rated oral health [19]. In this study, dental caries, self-perceived oral health and use of dental services remained associated with dental pain in the final model. Severe caries is the main cause of dental pain and subjective oral health measures, such as self-perceived oral health and dental pain, are strongly associated. These associations have already been reported by others authors in both adolescents [36] and adults [37]. Therefore, it can be suggested their mediator role on the possible harmful influence of individual and neighborhood social capital on dental pain. Notwithstanding, not only the prevalence of dental caries but also its severity has to be taken into account to explain its role on dental pain and to support the relationship between social capital and dental pain [38].

Contrary to findings reported in previous studies, individual socioeconomic characteristics, such as family income and sanitation conditions, were not associated with dental pain [11,39,40]. The high coverage of water supply and the financial governmental support programs for low-income families in the communities where the study was conducted may partially explain the discrepancies between our findings and the previous studies.

There are some limitations to our study. The number of clusters (neighborhoods) and units per cluster (individuals) can be considered weaknesses because of their influence on the study’s power. In order to compensate the limited number of individuals per cluster, data for all the age groups were analyzed simultaneously. Even though, age was considered as a covariate in the statistical analysis, which reduced its confounding effect, epidemiological studies in social capital and oral health usually considered specific age groups. Social capital meanings vary according to age and aggregating individual data from adolescents, adults and elderly to build the neighborhood social capital measure may affect its assessment. This bias was minimized by including similar proportions of each age group from the selected communities in the study. The proportion of each age group was similar across the neighborhoods, namely, around 60% of adolescents, 30% of adults and 10% of elderly. Another limitation is the cross-sectional design employed, which is directly related to limitations concerning causal inference and the occurrence of inverse causality, as some risk factors can be consequences of dental pain. This aspect is particularly relevant for some covariates, including utilization of dental care, time after the last dental visit and self-perceived oral health since they can precede dental pain as well as being consequences of dental pain. Similar to previous studies, these covariates were included in the multivariate logistic regression [15,16,19,20]. The choice to use census tracts to define neighborhoods can be criticized, as it is not necessarily related to the individual’s perception of their neighborhood. Finally, due to the high response rate it was not possible to estimate its variation among the neighborhoods.

From a public health policy perspective, policy makers may wish to know whether they should make interventions with regard to individuals or the places where they live. It is very likely that the answer is both, but that the strongest association observed between contextual social capital and dental pain suggests that priority should be placed on the latter. Although there is a claim to examine and tackle the ‘upstream’ social conditions that give rise to an unequal distribution of diseases [2], further evidence through prospective follow-up research into the influence of social capital is required to orientate dental public health policies, improve equity and reduce oral health inequalities.

## Conclusions

This study pointed out that contextual and individual social capital are independently associated with dental pain. The strongest association observed between neighborhood social capital and dental pain compared to individual social capital suggests that the effect of social context on dental pain is more important than individual social relationships.

## Competing interests

The authors declare that they have no competing interests.

## Authors’ contributions

BMS was involved in the planning, conception, design and conducting of the study. She carried out the field-work for data collection and drafted the manuscript. AMGV participated in the design of the study and drafted the manuscript. MVV contributed to the overall conceptualization and design of the study, performed the statistical analysis and drafted the manuscript. All authors have read and approved the final manuscript.

## Pre-publication history

The pre-publication history for this paper can be accessed here:

http://www.biomedcentral.com/1472-6831/13/2/prepub
